# Lectures on Diseases Nervous System

**Published:** 1876-03

**Authors:** 


LECTURES ON DISAESES OF THE NERVOUS SYSTEM. By Jerome K. Beawdery, M.D., Professor of Psycological Medicine and Diseases of the Nervous System and Medical Jurisprudence in the Missouri Medical College; Physician to St. Vincent's Institution for the Insane; late Superintendent of the St. Louis County Insane Asylum, etc. Reported by V. Biart, M.D. Revised and edited by the author. Philadelphia: J. B. Lippincott & Co. 1876.

This work of 476 pages is more particularly designed for the student and general practitioner who have no time to consult the more extended works upon the subjects of which it treats. "The aim," as the author expresses it, "being to present a thorough digest of the extensive field of medical literature on the subject of nervous diseases, and at the same time, so far as is consistent with a true portraiture of the maladies delineated, and a clear idea of their characteristic features, to avoid diffuseness of detail and description. The book may be had at publishers' prices, at Philips & Crew's, corner Peachtree and Marietta streets, Atlanta, Ga.

